# ^13^C^15^N: glucagon-based novel isotope dilution mass spectrometry method for measurement of glucagon metabolism in humans

**DOI:** 10.1186/s12014-022-09344-2

**Published:** 2022-05-19

**Authors:** Santosh Renuse, Linda M. Benson, Patrick M. Vanderboom, F. N. U. Ruchi, Yogesh R. Yadav, Kenneth L. Johnson, Benjamin C. Brown, Jane A. Peterson, Rita Basu, Daniel J. McCormick, Akhilesh Pandey, Ananda Basu

**Affiliations:** 1 Department of Laboratory Medicine and Pathology, Mayo Clinic, 200 First ST SW, Rochester, MN 55905 USA; 2 Center for Individualized Medicine, Mayo Clinic, Rochester, MN 55905 USA; 3 Mayo Genomics Facility-Proteomics Core, Mayo Clinic, Rochester, MN 55905 USA; 4 Division of Endocrinology, Center of Diabetes Technology, University of Virginia School of Medicine, Charlottesville, VA 22903 USA; 5 Department of Biochemistry and Molecular Biology, Mayo Clinic, Rochester, MN 55905 USA; 6 Department of Molecular Pharmacology and Experimental Therapeutics, Mayo Clinic, Rochester, MN 55905 USA; 7 Manipal Academy of Higher Education, Manipal, Karnataka 576104 India

**Keywords:** Stable isotope tracers, Type 1 diabetes, Oral glucose tolerance test, Insulin

## Abstract

**Background:**

Glucagon serves as an important regulatory hormone for regulating blood glucose concentration with tight feedback control exerted by insulin and glucose. There are critical gaps in our understanding of glucagon kinetics, pancreatic α cell function and intra-islet feedback network that are disrupted in type 1 diabetes. This is important for translational research applications of evolving dual-hormone (insulin + glucagon) closed-loop artificial pancreas algorithms and their usage in type 1 diabetes. Thus, it is important to accurately measure glucagon kinetics in vivo and to develop robust models of glucose-insulin-glucagon interplay that could inform next generation of artificial pancreas algorithms.

**Methods:**

Here, we describe the administration of novel ^13^C^15^N heavy isotope-containing glucagon tracers—FF glucagon [(Phe 6 ^13^C_9_,^15^N; Phe 22 ^13^C_9_,^15^N)] and FFLA glucagon [(Phe 6 ^13^C_9_,^15^N; Phe 22 ^13^C_9_,^15^N; Leu 14 ^13^C_6_,^15^N; Ala 19 ^13^C_3_)] followed by anti-glucagon antibody-based enrichment and LC–MS/MS based-targeted assays using high-resolution mass spectrometry to determine levels of infused glucagon in plasma samples. The optimized assay results were applied for measurement of glucagon turnover in subjects with and without type 1 diabetes infused with isotopically labeled glucagon tracers.

**Results:**

The limit of quantitation was found to be 1.56 pg/ml using stable isotope-labeled glucagon as an internal standard. Intra and inter-assay variability was < 6% and < 16%, respectively, for FF glucagon while it was < 5% and < 23%, respectively, for FFLA glucagon. Further, we carried out a novel isotope dilution technique using glucagon tracers for studying glucagon kinetics in type 1 diabetes.

**Conclusions:**

The methods described in this study for simultaneous detection and quantitation of glucagon tracers have clinical utility for investigating glucagon kinetics in vivo in humans.

**Supplementary Information:**

The online version contains supplementary material available at 10.1186/s12014-022-09344-2.

## Background

Post-absorptive and early post-prandial hyper-glucagonemia is a feature of both type 1 and type 2 diabetes and likely contributes to fasting and prandial hyperglycemia in these individuals [1–3]. In addition, there is inadequate plasma glucagon concentration in response to declining plasma glucose levels in the late post-prandial period increasing the risk for and delayed recovery from hypoglycemia [1, 4]. Similarly, plasma glucagon concentrations are suppressed during exercise in people with type 1 diabetes (T1D) thus increasing their risk for hypoglycemia [5]. However, the causes of these abnormalities are anticipated but not proven to be due to abnormal glucagon secretion. Circulating plasma glucagon concentration at any given point in time reflects a net balance between the rates of glucagon secretion from the α cells in the pancreatic islets of Langerhans, hepatic glucagon extraction if any, glucagon volume of distribution (VD), and systemic glucagon clearance/disappearance. In contrast to the C-peptide model that is available to measure insulin secretion in vivo [6, 7], no such model exists that measures glucagon secretion and kinetics. A better understanding of glucagon kinetics and its dysregulation in humans with diabetes that results in abnormalities in circulating plasma glucagon concentrations should lead to rational and informed therapeutic strategies to mitigate both hyperglycemia and hypoglycemia in these individuals.

There have been previous attempts to measure glucagon kinetics, clearance, and VD in animals [8–10] and in humans with [11, 12] and without diabetes [13, 14]. These have provided differing results that are due, at least in part, to variations in methods used, species tested, and glucagon assays utilized. However, rather than using specific glucagon tracers to measure glucagon kinetics, all of these experiments relied upon changes in plasma glucagon concentrations in the presence/absence of inhibition of endogenous glucagon secretion to infer glucagon secretion rates. Notably, none of these experiments conducted an in-depth analysis of glucagon kinetics in T1D. Deacon et al. [8] used antibodies specific to the C-terminal, N-terminal, and mid-region of glucagon molecule to examine glucagon metabolism in pigs. A few studies have described the detection of glucagon by LC–MS-based approaches [15, 16].

To circumvent the methodological limitations, we employed novel isotopically heavy glucagon tracers in combination with high resolution mass spectrometry-based assays to directly measure glucagon in plasma samples. Here, we describe anti-glucagon antibody-based enrichment of glucagon from plasma samples followed by targeted liquid chromatography-tandem mass spectrometry (LC–MS/MS) assays for absolute quantitation of endogenous and heavy tracers of glucagon. The limit of quantitation was measured at 1.56 pg/ml. This novel isotope dilution mass spectrometry assay has numerous applications for measuring glucagon secretion, post-hepatic glucagon appearance, glucagon volume of distribution and systemic glucagon clearance in the fasted and fed state in human research participants. The proposed method will also allow, for the first time, to precisely determine the key paracrine interaction, its dysregulation and help develop an explicit glucagon model for testing in the FDA-approved T1D simulator [17, 18] prior to clinical trials.

## Methods

### Chemicals and reagents

Reference pooled plasma for calibration curves was purchased from Innovative Research, Inc. (Catalog# IPLA-K2EDTA-29411, Novi, MI). Phosphate buffered saline (PBS) was purchased from Thermo (Catalog# AM9625, San Jose, CA), trifluoroacetic acid was purchased from Thermo Fisher Scientific (Catalog# 85183, Waltham, MA), zwittergent Z3-16 was purchased from CalBiochem (Catalog# CAS 2281-11-0, EMD Millipore, Billerica, MA), Dynabeads M-280 streptavidin was purchased from Invitrogen (Catalog# 11206D, Carlsbad, CA), formic acid was purchased from Thermo Scientific (Catalog# TS-28905, San Jose, CA) and acetonitrile was purchased from J.T. Baker (Catalog# 75-05-8, Phillipsburg, NJ). Leupeptin (Catalog#L2884) and aprotinin (Catalog#A6279) were purchased from Sigma-Aldrich (St. Louis, MO) and biotinylated rabbit polyclonal anti-glucagon antibody was purchased from Abcam (Catalog# ab48287, Cambridge, MA).

### Synthesis of isotopically labeled heavy glucagon tracers

All isotopically labeled synthetic glucagon tracers were synthesized using standard FMOC chemistry on a Liberty Blue peptide synthesizer (CEM Corp. Matthews, NC). A stable isotope labeled amino acid was incorporated into the sequence using Fmoc-Phenylalanine-OH ^13^C_9_,^15^N_1_ (Catalog# 651443), Fmoc-Leucine-OH ^13^C_6_^15^N (Catalog#593532), or Fmoc-Alanine-OH ^13^C_3_ (Catalog# 605131) (IsoTec, Inc., Miamisburg, OH). Peptides were cleaved using the CEM Razor cleavage module heated to 42 °C for 40 min. Cleavage cocktail was trifluoroacetic acid (Catalog# TX1275-3, Fisher Scientific, Hanover Park, IL), water, triisopropyl silane (Catalog# 233781, Sigma Aldrich, St. Louis, MO) and 3,6-Dioxa-1,8-octanedithiol (Catalog#D264925G, Fisher Scientific, Hanover Park, IL) (92.5%/2.5%/2.5%/2.5% v/v/v/v). Peptides were precipitated in cold methyl-t-butyl ether (Catalog# E1274, Fisher Scientific, Hanover Park, IL), washed, and dried for purification. Purification was achieved by reversed-phase HPLC using a Phenomenex Jupiter C_18_ column, 250 mm × 21.2 mm (Catalog# 00G-4057-P0, Torrance, CA), using a water/acetonitrile buffer system. Peptide masses were verified using 6230B time-of-flight mass spectrometer (Agilent Technologies Inc. Santa Clara, CA) interfaced with 1100 series high pressure liquid chromatography (HPLC) system (Agilent Technologies Inc. Santa Clara, CA). Primary stock standards were prepared at 1 mM concentration in dimethyl sulfoxide and stored at 4 °C. Net peptide content was determined by amino acid analysis.

### Preparation of infusate containing heavy glucagon tracers

Preparation of [^13^C^15^N] glucagon single dose vials: [^13^C^15^N] glucagon tracer was synthesized and stored in freezer desiccator (− 20 °C) in powder form. On the morning of preparation of single dose vials, the powder was placed at room temperature for one hour in a desiccator under a class II biosafety cabinet hood. Strict aseptic and sterile precautions were maintained throughout the process. 1% human serum albumin (HSA) solution was prepared by mixing 800 µl of 25% HSA with 19.2 ml sterile water in a sterile evacuated glass vial. Appropriate and measured amount of [^13^C^15^N] glucagon powder was mixed with 1% HSA solution to obtain 10 µg in each vial. Subsequently, each vial was tightly covered with sterile seals, transferred to lyophilization flasks, and covered with sterile blanket and placed in a −80 °C freezer until completely frozen. Three holes were made in the sterile seal of each vial using a sterile needle and the lyophilization flasks were quickly transferred to lyophilizer. Vials were then lyophilized until dry and sterile caps applied under the biosafety cabinet. All lyophilized vials containing the [^13^C^15^N] glucagon tracers were stored in desiccator in a − 20 °C freezer for future use (bolus FF and bolus FFLA). Aliquots of glucagon tracers were stored at pre-lyophilization (FF Pre and FFLA Pre), post-lyophilization (FF Post and FFLA Post).

Preparation of [^13^C^15^N] glucagon infusate: lyophilized [^13^C^15^N] glucagon (10 µg/vial) was reconstituted by adding 9.9 ml of sterile water and 0.1 ml of 5% HSA to achieve the final concentration of stock solution equivalent to 1 µg/ml. This was done in the morning of the research study under sterile and aseptic precautions in a class II biosafety cabinet. The solution was then mixed slowly to avoid frothing. The calculated amount of [^13^C^15^N] glucagon (based on subject body weight) was then withdrawn from the stock solution and mixed with required amount of normal saline and 0.4 ml of 25% HSA to achieve ~ 5% enrichment of glucagon. 1 ml of the diluted infusate sample was withdrawn into a Rohren tube and stored at − 80 °C to measure the concentration of [^13^C^15^N] glucagon in the infusate.

### Administration of heavy tracers to study participants and sample collection

All samples were collected after informed consent and approval by the Institutional Review Board (IRB) at University of Virginia. A 20-gauge IV catheter was inserted into a forearm vein for the infusion of glucagon tracers and another IV catheter was inserted in a retrograde fashion into a hand vein of the contralateral arm and the hand placed in a heated box, whose temperature was regulated at 55 °C, for drawing arterialized venous blood samples throughout the study. Blood samples were aliquoted into EDTA tubes, centrifuged for 15 min and separated plasma was then aliquoted and stored at − 80 °C until analyses. Samples from non-diabetic and type 1 diabetes patient were collected at predefined time intervals.

### Sample processing for enrichment of glucagon tracers

All steps were performed on ice unless indicated. The streptavidin-magnetic beads were resuspended in storage buffer with brief vortex and storage buffer was discarded using magnetic rack. The beads were washed twice with PBS/0.03% CHAPS. Antibody was added to magnetic beads to a final volume ratio of 6:75 and incubated at 4 °C/1400 rpm for 2 h. The antibody-beads were then washed thrice with PBS/0.03% CHAPS and stored on ice until needed. Calibration curve samples were prepared using reference human plasma spiked-in with light and heavy tracers of glucagon as shown in Table 1. Quality control samples were prepared at high (light glucagon—80 pg/ml, heavy tracers 40 pg/ml), medium (light glucagon—26.67 pg/ml, heavy tracers 13.33 pg/ml) or low (light glucagon—8.88 pg/ml, heavy tracers—4.44 pg/ml) concentrations as shown in Table 2. The infusate samples were prepared for FF [(Phe 6 ^13^C_9_,^15^N; Phe 22 ^13^C_9_,^15^N)—glucagon] and FFLA [(Phe 6 ^13^C_9_,^15^N; Phe 22 ^13^C_9_,^15^N; Leu 14 ^13^C_6_,^15^N; Ala 19 ^13^C_3_)—glucagon] in pooled reference plasma by using 1/100 (Dilution A), 1/500 (Dilution B), and 1/2000 (Dilution C) dilutions as shown in Table 3 for pre, post and bolus FF and FFLA glucagon.

**Table 1:** Calibration curve concentration range

	Std 7 (pg/ml)	Std 6 (pg/ml)	Std 5 (pg/ml)	Std 4 (pg/ml)	Std 3 (pg/ml)	Std 2 (pg/ml)	Std 1 (pg/ml)
Light glucagon	200	100	50	25	12.5	6.25	3.125
FF glucagon	100	50	25	12.5	6.25	3.125	1.56
FFLA glucagon	100	50	25	12.5	6.25	3.125	1.56

**Table 2:** List of plasma quality control samples

QC standard	QC1 (pg/ml)	QC2 (pg/ml)
Light glucagon	26.67	80
FF glucagon	13.33	40
FFLA glucagon	13.33	40

**Table 3:** Dilution scheme for glucagon tracers (FF and FFLA) infusates

Infusate 0.05 µg/ml Expected conc	Dilution A 1/100 Expected conc. (pg/ml)	Dilution B 1/500 Expected conc.	Dilution C 1/2000 Expected conc.
FF pre	465	93	23.25
Bolus FF	465	93	23.25
FF post	465	93	23.25
FFLA pre	465	93	23.25
Bolus FFLA	465	93	23.25
FFLA post	465	93	23.25

Plasma samples (0.5 ml) from subjects infused with heavy glucagon tracers were thawed and centrifuged for 10 min at 4000×*g* at 4 °C. The enrichment was carried out in 2 ml 96-well plates. Briefly, all samples (specimens, calibration curve, quality control and infusates) were diluted 1:1 with PBS/0.03% CHAPS with protease inhibitors, mixed at 1350 rpm for 1 min at 4 °C. F22 glucagon internal standard was added to each sample at 50 pg/ml except the plasma only control. The plate was centrifuged briefly to 1000×*g* at 4 °C. 5 µg of antibody was added to each sample and incubated by shaking overnight at 1350 rpm at 3 °C. After incubation, the supernatant was collected, and the beads were washed three times using PBS/0.03% CHAPS. Beads were then washed with PBS/0.002% zwittergent 3–16 followed by a wash using 50 mM NH4Ac (pH 4.6). Glucagon was eluted from the beads using extraction buffer (aqueous 0.2% formic acid, 0.1% trifluoroacetic acid, 0.002% zwittergent 3–16), collected in autosampler vials and immediately analyzed by LC–MS/MS.

### Development of targeted SIM and parallel reaction monitoring (PRM) assay

Targeted SIM scan followed by parallel reaction monitoring (PRM) analysis was performed on a Q-Exactive Plus Orbitrap mass spectrometer interfaced with an Ultimate 3000 RSLCnano system (Thermo Scientific, Bremen, Germany). Extracted glucagon samples were loaded on a trap column (Halo ES-C_18_; 2.7 µm, 160 Å, 0.33 µl) at a flow rate of 40 µl/min for 5 min followed by separation using an analytical column (PicoFrit Agilent Poroshell 120 EC-C_18_; 2.7 µm; 100 µm × 30 cm) at 400 nl/min flow rate with a 17 min linear gradient from 5% solvent B (0.2% formic acid/80% acetonitrile/10% IPA/10% water) to 70% solvent B (6 min to 23 min) with a total run time of 33 min. Data acquisition parameters included MS1 SIM scan at 875 m/z with a isolation width of 12 m/z followed by ddMS2/PRM analysis of m/z 875 with a isolation width of 12 m/z using higher energy collision dissociation (HCD) normalized collision energy (NCE) of 24. The targeted SIM scan parameters were: Orbitrap resolution of 70,000@400 m/z, AGC target value of 5 × 10^5^, injection time of 300 ms while PRM scan parameters were: Orbitrap resolution of 35,000@400 m/z, AGC target value of 5 × 10^5^, injection time of 300 ms. The calibration curve, quality control and infusate samples were analyzed prior to analysis of subject specimens.

### Absolute quantitation of glucagon tracers

The raw mass spectrometry data was processed using Xcalibur Quan Browser (version 3.0) (ThermoFisher Scientific, San Jose, CA). A processing method was prepared with default integration algorithm—ICIS, 7 smoothing points, baseline window of 40, area noise factor of 5 and a peak noise factor of 10. Samples were integrated using the automatic functions and peak integration for light glucagon, internal standard (F22 glucagon) and heavy glucagon tracers (FF and FFLA glucagon) (Table 4) were assessed for consistency. Calibration curves were generated using control plasma spiked-in with light, FF and FFLA glucagon at 7 concentration levels (Table 1) and 50 pg/ml of internal standard (F22 glucagon). The concentration of heavy glucagon tracers was determined using equation from respective standard curves. The peak area results were exported, and glucagon kinetics determined from subject specimens.

**Table 4:** List of precursor and product ions considered for glucagon quantitation

Targeted-SIM	Label	m/z ions
Light glucagon	871.4214_MS1	871.41; 871.66
F22 (IS)	873.6634_MS1	873.67; 873.92; 874.17
FF	876.4232_MS1	876.17; 876.42; 876.68
FFLA	878.4269_MS1	878.68; 878.93; 879.18
PRM		
Glucagon (light)	871.4214_MS2	1002.14 (b25^3+^); 1002.47 (b25^3+^); 1039.83 (b26^3+^); 1040.17 (b26^3+^); 1083.51 (b27^3+^); 1083.85 (b27^3+^)
F22 (IS)	873.6634_MS2	1005.48 (b25^3+^); 1005.82 (b25^3+^); 1043.18 (b26^3+^); 1043.51 (b26^3+^); 1086.86 (b27^3+^); 1087.19 (b27^3+^)
FF	876.4232_MS2	1008.82 (b25^3+^); 1009.16 (b25^3+^); 1046.85 (b26^3+^); 1047.18 (b26^3+^); 1090.20 (b27^3+^); 1090.87 (b27^3+^)
FFLA	878.4269_MS2	1012.17 (b25^3+^); 1012.84 (b25^3+^); 1049.86 (b26^3+^); 1050.19 (b26^3+^); 1093.88 (b27^3+^); 1094.21 (b27^3+^)

### Assay validation

Intra-day and inter-day accuracy and precision were measured on three separate days. Each batch included a set of calibration standards and three replicates of reference plasma quality control samples spiked in with light/heavy tracers of glucagon at medium and high concentration (Table 2**)**.

### Systemic glucagon appearance and disappearance rate calculation

The rates of systemic glucagon appearance and disappearance were calculated by the isotope dilution technique based on non-steady state substrate turnover method as originally described for calculating endogenous glucose turnover by Steele et al. [19–21]. The method has subsequently been refined and applied using single isotope (tracer), dual isotope and triple isotope methods to estimate fasting and post prandial glucose [22–24] metabolism by using glucose isotopes, and endogenous cortisol metabolism [25] by using cortisol isotopes. Similar principles were applied to estimate endogenous glucagon metabolism (appearance, disappearance) using FF and FFLA glucagon as the glucagon isotopes (tracers).

1 $$Ra_{glucagon} = \left( {\left[ {F _{FFglucagon} /TTR _{FFglucagon} } \right] {-} F _{FFglucagon} {-}F _{FFLAglucagon} } \right) - \{ (V x C \times \left( {dTTR/dT} \right)/mean TTR\} ;$$

2 $$Rd_{glucagon } = \left( {Ra_{glucagon } + F_{FFglucagon} + F_{FFLAglucagon} } \right) {-} \left( {V\left( {dC/dT} \right)/ TBW} \right);$$

where *Ra*_*glucagon*_ is rate of appearance of glucagon, *F*_*FF glucagon*_ is rate of infusion of FF glucagon, *F *_*FFLA glucagon*_ is rate of infusion of FFLA glucagon, *TTR *_*FF glucagon*_ is the tracer tracee ratio of FF glucagon to total glucagon concentrations in plasma, *V* is the volume of distribution of glucagon, *C* is the concentration of total glucagon, *T* is the time (in minutes), *Rd*_*glucagon*_ is the rate of disappearance of glucagon, *d* is the integral of measures over time and *TBW* is the total body weight of the individual. FF glucagon decay following a bolus injection followed a linear single compartment model and was used to calculate glucagon volume of distribution—*V*, by non-linear least-squares regression analysis using Kinetica 5.1 SP1 (Thermo Fisher Scientific). This was calculated to be 40 ± 1.4 ml/kg total body weight in a cohort of 9 healthy subjects and 10 subjects with type 1 diabetes.

Plasma glucagon concentration (endogenous + glucagon tracer) was measured by standardized and conventional ELISA technique (Mercodia, Uppsala, Sweden). Intra-assay CVs were 6.7% and 7.8% at 65 and 183 pg/ml respectively. Inter-assay CVs were 7.5%, 4.0% and 2.9% at 62, 144 and 84 pg/ml respectively. The assay specificity is 100% for human glucagon with < 0.1% to oxyntomodulin and none to human insulin, proinsulin, SRIF or C-peptide.

## Results

**Fig. 1:**
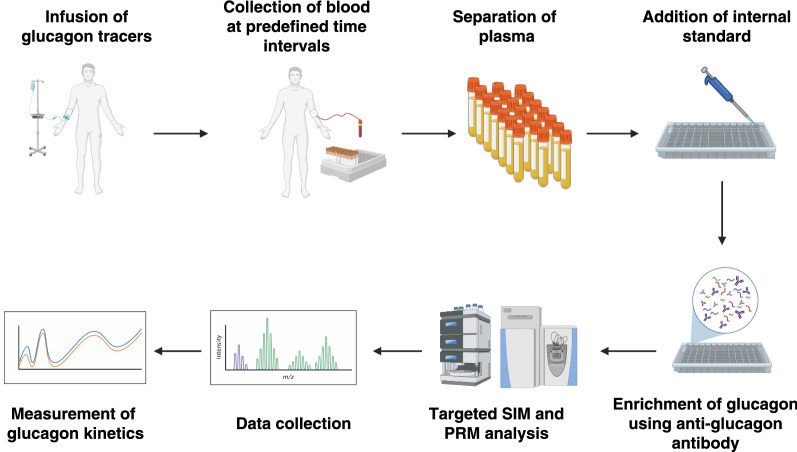
A schematic workflow for targeted LC–MS/MS analysis of glucagon tracers from clinical samples. Subjects were infused with stable isotopically labeled heavy tracers of glucagon followed by blood collection at specified time intervals. The plasma samples were then subjected to anti-glucagon antibody-based enrichment of endogenous and heavy glucagon tracers. PRM-based targeted LC–MS/MS analysis was carried using high resolution Q-Exactive Orbitrap mass spectrometer for measurement of glucagon kinetics

Here, we describe the synthesis of novel isotopically heavy labeled glucagon as tracers for measurement of glucagon kinetics in clinical samples. We deployed anti-glucagon antibody-based enrichment of glucagon followed by high resolution targeted mass spectrometry for detection and quantitation of glucagon as shown in Fig. 1. The method described in this study can be used for simultaneous quantitation of endogenous glucagon and isotopically labeled heavy tracers.

### Synthesis of novel heavy isotopically labeled glucagon as tracers

We designed and synthesized non-radioactive (i.e. stable) isotopes of human glucagon which were infused into human subjects without any adverse effects [26]. These heavy tracers include FF- glucagon: (Phe 6 ^13^C_9_,^15^N_1_; Phe 22 ^13^C_9_,^15^N_1_) and FFLA-glucagon: (Phe 6 ^13^C_9_,^15^N; Phe 22 ^13^C_9_,^15^N; Leu 14 ^13^C_6_,^15^N_1_; Ala 19 ^13^C_3_). The positions of the isotope-labeled residue replacements (bold underline) at 6, 22 for FF-glucagon and 6, 14, 19, 22 for FFLA-glucagon are shown: H2N-HSQGT**F**TSDYSKY**L**DSRR**A**QD**F**VQWLMNT-OH. We also synthesized stable isotope labeled (SIL) glucagon with a single heavy label on phenyl alanine as F22 (Phe 22 ^13^C_9_,^15^N_1_) to be used as an internal standard. SIL peptides are commonly used as internal standards in targeted proteomics applications including immuno-precipitation (IP)-MRM/PRM as normalization controls for monitoring sample to sample variations in ionization efficiency and, in some cases, enzymatic digestion. We carried out IP-PRM experiments using synthetic glucagon spiked-into reference pooled plasma for the determination of limit of detection and limit of quantitation.

### Anti-glucagon antibody-based enrichment of glucagon tracers

**Fig. 2:**
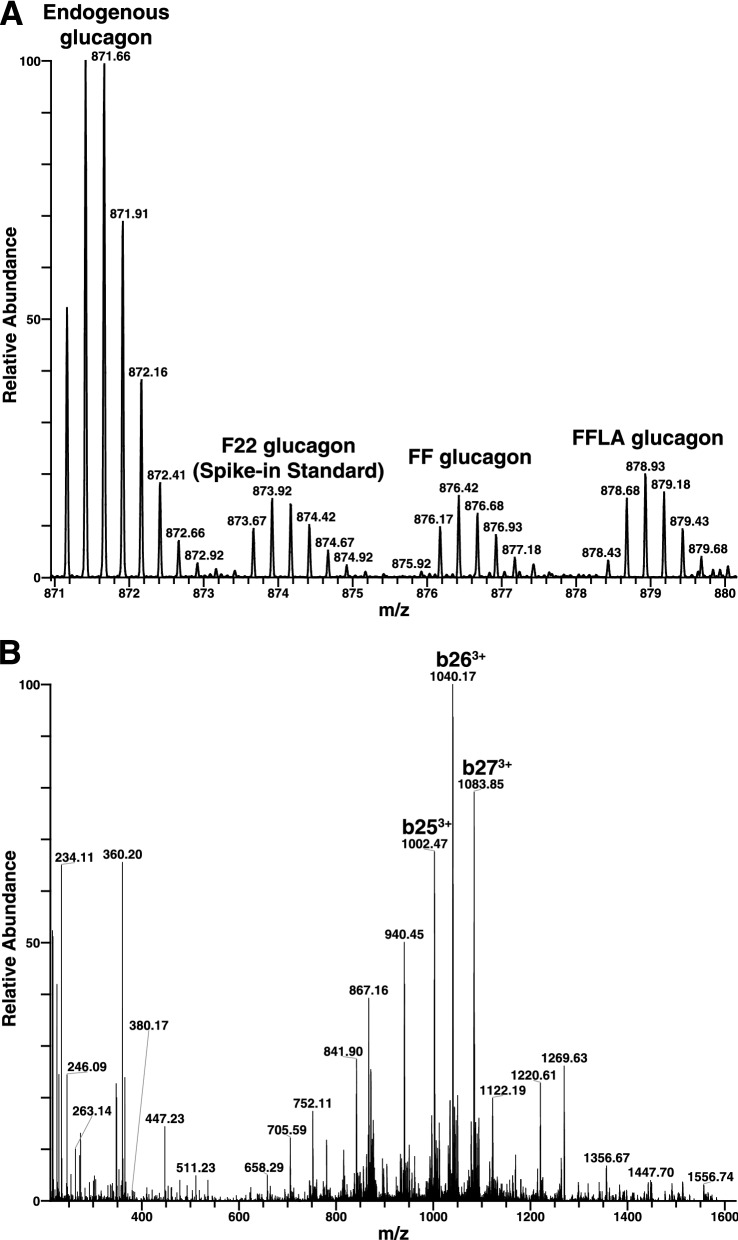
Representative mass spectrum showing targeted wide SIM scan (869–881) MS1 precursor ions for light glucagon, IS (F22 glucagon), FF glucagon and FFLA glucagon (**A**). Representative PRM scan showing abundant b-ions (b25^3+^, b26^3+^ and b27^3+^) (**B**). Inset shows respective b ions for endogenous glucagon, F22 glucagon (spike-in standard), FF glucagon and FFLA glucagon

Plasma samples were processed for enrichment of endogenous glucagon and heavy tracers using anti-glucagon antibody. Briefly, a biotinylated rabbit polyclonal antibody from Abcam (Catalog# 48287) was incubated with the plasma containing heavy glucagon tracers overnight at 4 °C. The antibody-antigen complexes were then purified using streptavidin magnetic beads. The peptides were released using 0.1% trifluoroacetic acid in 0.002% Z-316. The eluted peptides were then analyzed on a Q-Exactive Orbitrap mass spectrometer in targeted SIM scan as well as wide window PRM modes (Fig. 1). The 12 m/z window (m/z 869–871) was chosen to cover precursor ions of all four glucagon species (endogenous light Glucagon, F22, FF and FFLA glucagon tracers). The representative MS1 and MS/MS spectra are shown in Fig. 2A, B, respectively. The inset in Fig. 2B shows a magnified view of the MS/MS spectrum from m/z 1000–1100 showing b25^3+^, b26^3+^ and b27^3+^ ions corresponding to light glucagon, IS, FF glucagon and FFLA glucagon, respectively (Additional file 1: Fig. S1). The data analysis and absolute quantitation was carried out using Xcalibur Quan Browser considering precursor and product ions (Table 3).

### Limit of detection

**Fig. 3:**
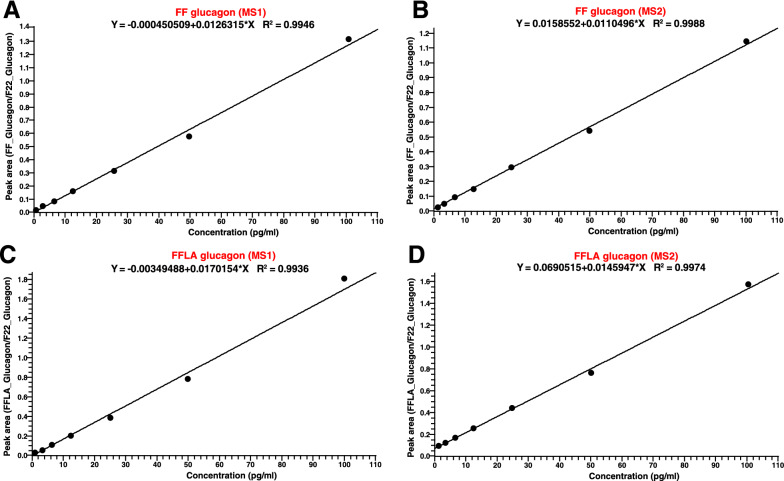
Calibration curves for quantitation of glucagon tracers in human plasma. The area ratio of glucagon tracers (FF and FFLA glucagon) to IS (F22 glucagon) was determined from the extracted ion chromatograms (EIC) at MS1 and MS2 levels. For calibration curve of FF glucagon, the combined EIC of m/z 876.17; 876.42; 876.68 at MS1 (**A**) and m/z 1008.82; 1009.16; 1046.85; 1047.18; 1090.20; 1090.8 at MS2 (**B**) was used while For FFLA glucagon, the combined EIC of m/z 878.68; 878.93; 879.18 at MS1 (**C**) and m/z 1012.17; 1012.84; 1049.86; 1050.19; 1093.88; 1094.21 at MS2 (**D**). The area ratios were plotted against concentration (range: 1.56–100 pg/ml)

The analyte (either FF glucagon or FFLA glucagon) to spike-in standard (F22 glucagon) ratios were used for quantitation analysis. Glucagon calibration curve samples were prepared by spiking glucagon light and heavy peptides in 500 µl of reference pooled plasma. The concentrations of FF and FFLA were spiked-in at 1.56, 3.12, 6.25, 12.5, 25, 50, 100 pg/ml while light glucagon was at 3.12, 6.25, 12.5, 25, 50, 100 and 200 pg/ml (Table 1). The IS F22 glucagon was spiked in at 50 pg/ml. Figure 3 shows calibration curve for FF glucagon at MS1 (Fig. 3A) and MS2 (Fig. 3B) levels while Fig. 3C and Fig. 3D show calibration curves for FFLA glucagon at MS1 and MS2 levels, respectively. Calibration curves were prepared using dilution series in triplicates and a linear regression analysis was carried out. The Pearson correlation coefficient (r) was observed to be > 0.99 for both FF and FFLA glucagon. The LOD was defined as the lowest concentration at which the peak for the analyte is detected with an S/N ratio of 3 and the mean result of the lowest standard is 3 SD above the blank results. LOQ was established as the lowest concentration that met the criteria of precision (CV ≤ 25%) and accuracy (RE ≤ 25%).

### Assay validation

Intra-day accuracy and precision were measured in triplicates followed by inter-day accuracy and precision measurements on three separate days. Each batch included a set of calibration standards and three replicates of reference plasma QC samples spiked-in with light/heavy tracers of glucagon. QC standards were prepared at two different concentration levels—13.3 pg/ml (QC1) and 44 pg/ml (QC2) of synthetic light and heavy (FF and FFLA) glucagon in addition to F22 glucagon as an internal standard. The calibration standards and QC samples were subjected to IP-PRM analyses and quantitation of glucagon tracers. The intra-day and inter-day precision were observed to be < 16% for FF glucagon and < 23% for FFLA glucagon (Table 5).

**Table 5:** Intra-day (n = 3) and inter-day (n = 3) precision measurements of heavy tracers of glucagon spiked-in to pooled reference plasma

FF glucagon Conc. (pg/ml)	Intra-day precision, CV (%)	Inter-day precision, CV (%)
13.3	5.2	15.9
40	5.6	13.6

FFLA glucagon Conc. (pg/ml)	Intra-day precision, CV (%)	Inter-day precision, CV (%)
13.3	3.9	22.9
40	4.9	18.9

### Glucagon turnover in type 1 diabetes

**Fig. 4:**
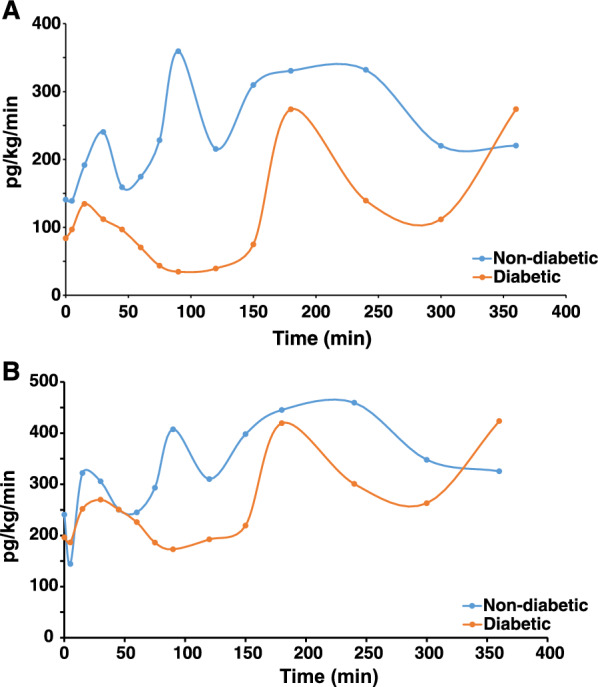
Glucagon kinetic measurements from clinical samples using targeted mass spectrometry. Postprandial rates of appearance (**A**) and disappearance (**B**) of glucagon in a non-diabetic (blue) and diabetic (orange) participant applying isotope dilution technique with isotopically heavy glucagon tracers

The optimized targeted assay was then applied to measure glucagon turnover in study participants with and without type 1 diabetes. Participants with or without type 1 diabetes were infused with heavy tracers of glucagon and arterialized venous samples were drawn periodically for measurements of ^13^C^15^N glucagon. The samples were analyzed by LC–MS/MS for measuring glucagon concentration as described above. The measured concentrations were then used for calculation of postprandial rates of appearance (Fig. 4A) and disappearance (Fig. 4B) of glucagon using non-steady-state equation applying isotope dilution technique. Representative examples of turnover data from a non-diabetic (blue) and a type 1 diabetic (orange) participant is shown.

## Discussion

We have demonstrated successful application of the isotope dilution technique to estimate glucagon turnover directly in vivo in humans with and without type 1 diabetes. The process included ex vivo synthesis of non-radioactive stable isotopes of glucagon, its purification, preparation of infusates and administration into human research participants after IRB approval, followed by accurate, sensitive, and precise analyses of plasma samples using tandem mass spectrometry. We applied the optimized assay for calculation and estimation of glucagon kinetics in plasma with estimation of systemic glucagon turnover in participants with and without type 1 diabetes [27]. To the best of our knowledge, this is the first ever use of this technique in humans to help in our understanding of glucagon physiology (secretion, clearance, volume of distribution) in health and disease. Avoidance of use of radioactive labelled glucagon significantly enhanced the safety and viability of its use in humans. Further, the structure and composition of glucagon, i.e., its single chain 29 amino acids peptide sequence devoid of any disulfide bonds made this a feasible approach to understand glucagon physiology and pathophysiology in type 1 diabetes where major abnormalities in circulating glucagon concentrations occur during various free-living conditions, e.g., fasting, postprandial and exercise. A better understanding of the reasons why glucagon concentrations differ in diabetes (changes in glucagon secretion, its clearance and/or volume of distribution) will help us design informed rational therapeutic approaches to normalize glucose control in these individuals.

It is important to note that glucagon kinetics (appearance, disappearance, clearance and volume of distribution) cannot be determined using only endogenous glucagon concentrations without the use of glucagon tracers/isotopes. This is because plasma glucagon concentrations measured at any given time reflect the net balance of rate of glucagon entering the circulation (glucagon appearance), rate of glucagon leaving the circulation (glucagon disappearance), its volume of distribution (Vd) and metabolic clearance rate. The use of glucagon isotope that we can measure accurately (by LC–MS/MS) and distinguish from endogenous glucagon is essential to estimate endogenous glucagon turnover. This principle also holds true for estimation of turnover of other endogenous substrates and hormones e.g., glucose, cortisol, free fatty acids, lactate and ketone bodies, where corresponding isotopes have been utilized for measuring similar parameters for the respective endogenous substances.

Further, isotope dilution method is based on the principle that one should be able to measurably distinguish the isotope of the substrate from the endogenous substrate. Here, we have fulfilled that premise by using LC–MS/MS to measure the glucagon isotope and ELISA to measure total glucagon (endogenous + isotope). The ELISA method cannot distinguish the glucagon isotope from endogenous glucagon and therefore measures all glucagon in plasma. We are thereafter able to calculate endogenous glucagon concentrations by subtracting the glucagon isotope concentration from the total glucagon concentrations. The tracers behave the same way as endogenous glucagon since the 29 amino acid backbone of the glucagon isotope is identical to that of endogenous glucagon molecule.

The end point of  ~ 350 min was selected to measure postprandial glucagon turnover after a mixed meal. Based on our extensive prior experience of measuring meal carbohydrate turnover in individuals with and without type 1 diabetes, a duration of ~ 6 h after meal consumption is sufficient to define postprandial turnover of substrates (e.g., carbohydrates, fats and protein) and the relevant hormones (i.e., glucagon).

Finally, mechanisms of action of novel therapeutic strategies that are currently being investigated (glucagon receptor antagonists) or have already been approved (SGLT2 inhibitors) could be better probed, as they relate to pancreatic α cell function, by applying this technique. For example, plasma glucagon concentrations increase during SGLT2 inhibitor therapy for diabetes, contributing, at least in part to the three- to fourfold increased risk of ketoacidosis in these individuals. The reasons for this increase are currently unknown. Using the glucagon isotope dilution technique would help determine if the rise in circulating glucagon concentrations are related to increase in pancreatic glucagon secretion, reduction in systemic glucagon clearance, change in glucagon volume of distribution or a combination of all or some of the above mechanisms. Once understood, rational therapeutic approaches could be fashioned to mitigate this rise in glucagon concentrations thus reducing the risk for ketoacidosis, a potentially problematic acute complication of diabetes.

## Conclusions

A novel isotope dilution mass spectrometry method using ^13^C^15^N-glucagon was developed using an anti-glucagon antibody-based approach. The optimized targeted assay developed in this study was applied to measurement of glucagon turnover in human plasma. Understanding glucagon kinetics is important in diabetes for better management and therapeutics. We envision that the method describe in this study will pave the way for monitoring of glucagon levels in individuals with diabetes.

## Supplementary Information

**Additional file 1: Figure S1.** Representative MS/MS spectra showing mapped b-ions for ions for endogenous glucagon (pink color), F22 glucagon (spike-in standard) (blue color), FF glucagon (brown color) and FFLA glucagon (green color) for respective b25^3+^ ions (panel A), b26^3+^ ions (panel B) and b^27^3 + ions (panel C).

## Data Availability

The mass spectrometry proteomics data have been deposited to the ProteomeXchange Consortium via the PRIDE partner repository [28] with the dataset identifier PXD028715.

## Abbreviations

VD: Volume of distribution
T1D: Type 1 diabetes
LC–MS/MS: Liquid chromatography–tandem mass spectrometry
