# Supplementary figures and images for: 13C15N: glucagon-based novel isotope dilution mass spectrometry method for measurement of glucagon metabolism in humans
Source: Clin Proteomics. 2022 May 19;19:16. doi: 10.1186/s12014-022-09344-2 (PMC9118570; doi:10.1186/s12014-022-09344-2)

**A**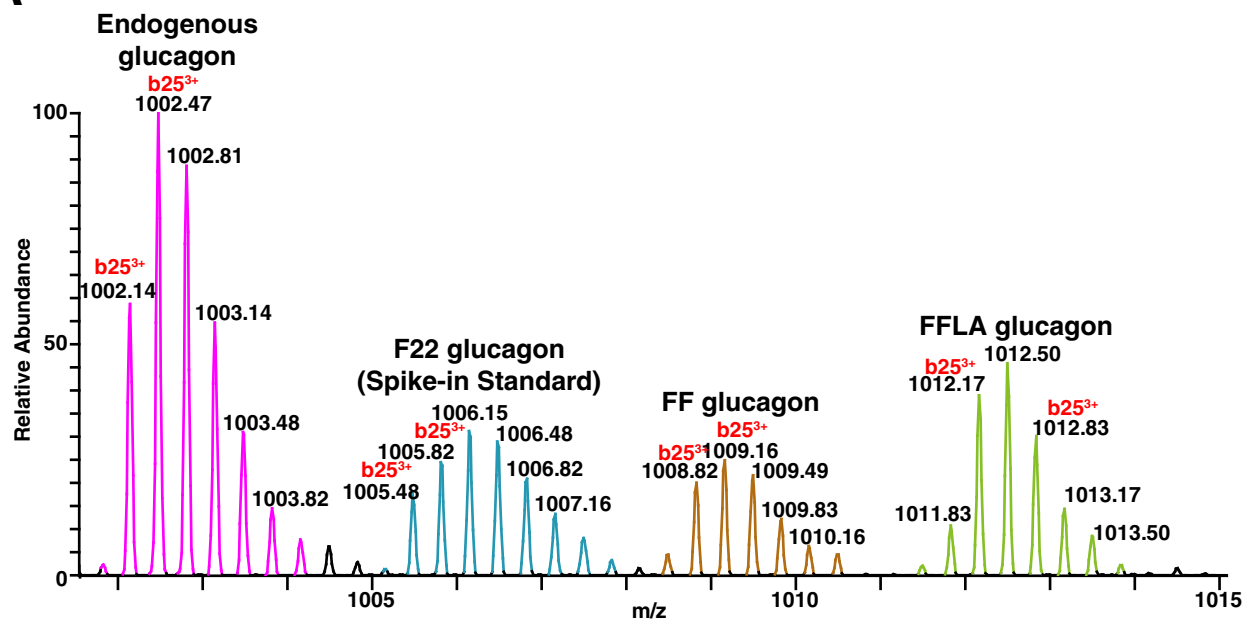**B**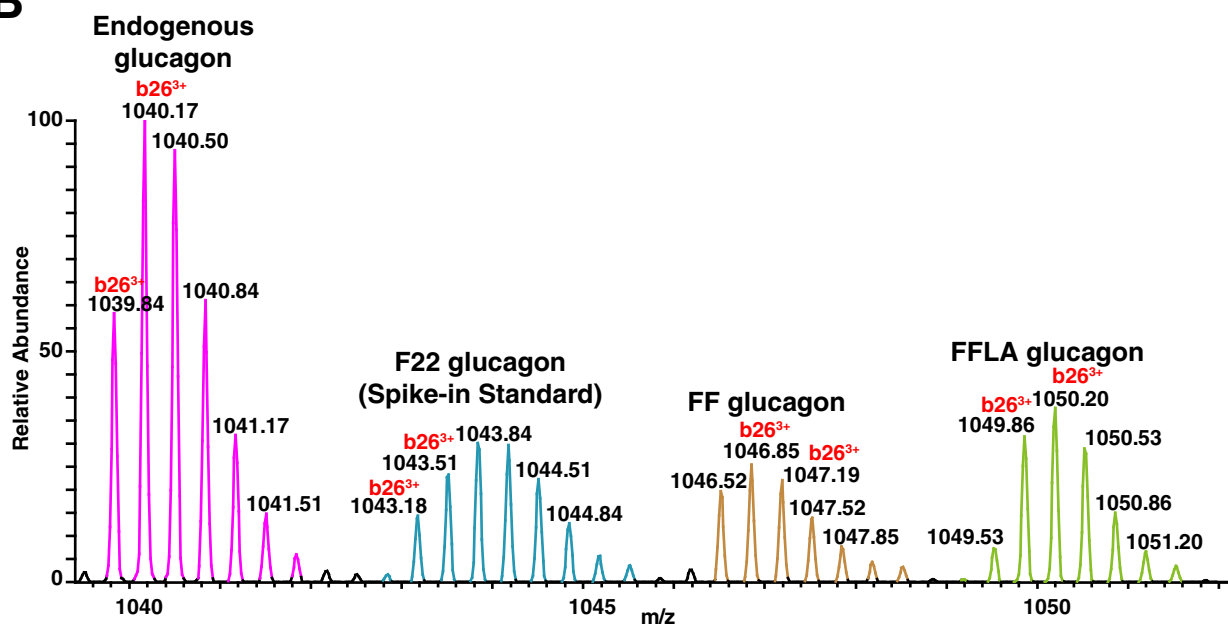**C**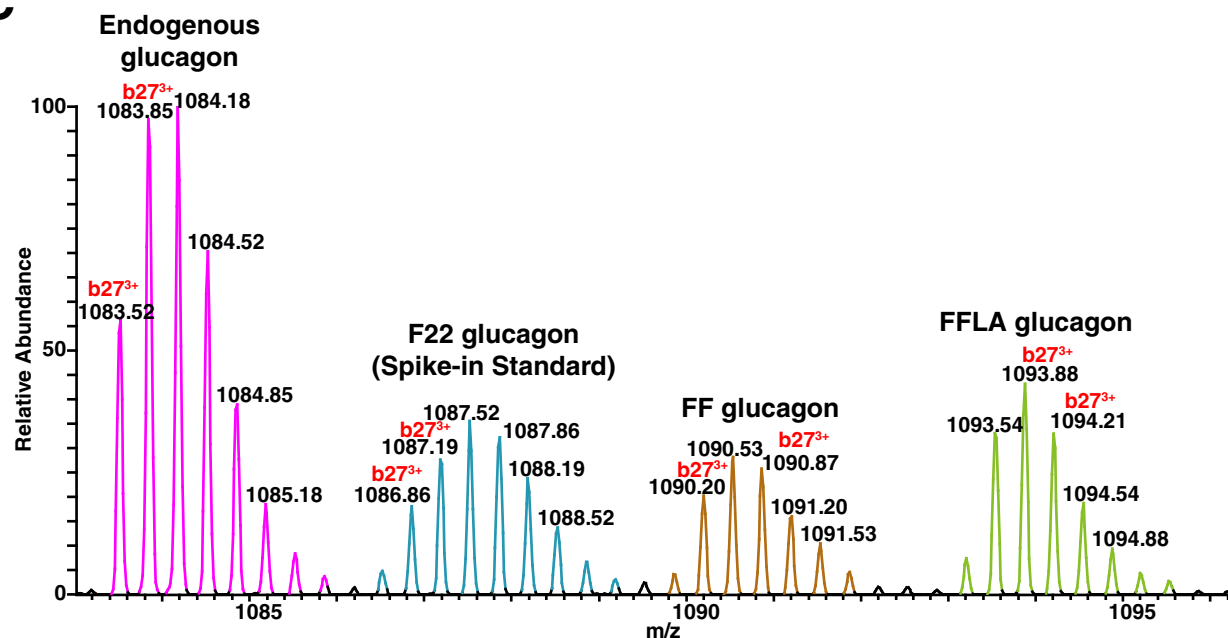

Supplement: Supplementary file 1 — Additional file 1: Figure S1. Representative MS/MS spectra showing mapped b-ions for ions for endogenous glucagon (pink color), F22 glucagon (spike-in standard) (blue color), FF glucagon (brown color) and FFLA glucagon (green color) for respective b253+ ions (panel A), b263+ ions (panel B) and b273 + ions (panel C). [file 12014_2022_9344_MOESM1_ESM.pdf]
